# Rescuing eGFP-Tagged Canine Distemper Virus for 40 Serial Passages Separately in Ribavirin- and Non-Treated Cells: Comparative Analysis of Viral Mutation Profiles

**DOI:** 10.3389/fcimb.2021.746926

**Published:** 2021-09-16

**Authors:** Fuxiao Liu, Ning Wang, Jiahui Lin, Qianqian Wang, Yilan Huang, Youming Zhang, Hu Shan

**Affiliations:** ^1^College of Veterinary Medicine, Qingdao Agricultural University, Qingdao, China; ^2^State Key Laboratory of Microbial Technology, Shandong University, Qingdao, China

**Keywords:** rCDV-eGFP, ribavirin, quasispecies, next-generation sequencing, SNM, high-fidelity

## Abstract

Due to lacking a proofreading mechanism in their RNA-dependent RNA polymerases (RdRp), RNA viruses generally possess high mutation frequencies, making them evolve rapidly to form viral quasispecies during serial passages in cells, especially treated with mutagens, like ribavirin. Canine distemper virus (CDV) belongs to the genus *Morbillivirus*. Its L protein functions as an RdRp during viral replication. In this study, a recombinant enhanced green fluorescence protein-tagged CDV (rCDV-eGFP) was rescued from its cDNA clone, followed by viral identification and characterization at passage-7 (P7). This recombinant was independently subjected to extra 40 serial passages (P8 to 47) in ribavirin- and non-treated cells. Two viral progenies, undergoing passages in ribavirin- and non-treated VDS cells, were named rCDV-eGFP-R and -N, respectively. Both progenies were simultaneously subjected to next-generation sequencing (NGS) at P47 for comparing their quasispecies diversities with each other. The rCDV-eGFP-R and -N showed 62 and 23 single-nucleotide mutations (SNMs) in individual antigenomes, respectively, suggesting that the ribavirin conferred a mutagenic effect on the rCDV-eGFP-R. The spectrum of 62 SNMs contained 26 missense and 36 silent mutations, and that of 23 SNMs was composed of 17 missense and 6 silent mutations. Neither the rCDV-eGFP-R nor -N exhibited nonsense mutation in individual antigenomes. We speculate that the rCDV-eGFP-R may contain at least one P47 sub-progeny characterized by high-fidelity replication in cells. If such a sub-progeny can be purified from the mutant swarm, its L protein would elucidate a molecular mechanism of CDV high-fidelity replication.

## Introduction

Canine distemper is a severe infectious disease, affecting a broad variety of domestic and wild carnivores (McCarthy et al., 2007). Its etiological agent is canine distemper virus (CDV), also known as canine morbillivirus, classified into the genus *Morbillivirus* in the family *Paramyxoviridae*. Canine distemper virions are enveloped and pleomorphic particles containing single-stranded RNA with negative polarity. CDV generally has a genome length of 15 690 nucleotides (nt), following the ‘‘rule of six’’, required for the efficient replication between the genome and antigenome (Kolakofsky et al., 2005).

A CDV genome contains six transcriptional units, independently encoding six structural proteins (N, P, M, F, H and L proteins). Additionally, CDV codes for two nonstructural proteins (V and C proteins). The V protein is expressed *via* an RNA editing strategy from a P gene transcription unit (Mahapatra et al., 2003). All genes are arranged in an order of 3’-N-P/V/C-M-F-H-L-5’ in the CDV genome. Six open reading frames (ORFs) are separated by untranslated regions (UTRs) with variable lengths. The L protein is an RNA-dependent RNA polymerase (RdRp), which is the largest of the virus proteins and is also the least abundant. It is assumed to carry all activities necessary for genomic RNA transcription and replication, as well as to be able to cap, methylate and polyadenylate viral mRNAs (Barrett et al., 2006). The morbilliviral L protein can only perform its function as the RdRp, when it associates with its co-factor, the P protein.

RNA viruses generally possess relatively high mutation frequencies in nature, mainly attributed to the lack of proofreading mechanisms in their RdRps (Mandary et al., 2019). Morbilliviruses are no exception (Liu et al., 2016). Due to the low-fidelity characteristics of morbilliviral RdRp, random mutations would unavoidably occur during replication between the genome and antigenome. Single-nucleotide mutations (SNMs) are theoretically restricted only in a small number of sites during the first few passages of morbillivirus in cells, whereas the continuous proliferation of progenies undoubtedly results in more complex mutant spectra, namely viral quasispecies. Viral quasispecies can be defined as the mutant distributions that are generated upon replication of RNA viruses *in vitro* and *in vivo* (Andino and Domingo, 2015). Owing to the absence of proofreading mechanisms in their RdRps, RNA viruses can be regarded as ideal models for experimentally addressing key questions in their dynamics of replication and evolution (Arbiza et al., 2010; Sardanyes and Elena, 2011).

It is not easy to determine accurately the quasispecies diversity in viral progenies. For example, Sanger sequencing as a conventional method is incapable of creating a large-scale dataset to identify all SNMs in viral mutants (Wu et al., 2016). The advent of next-generation sequencing (NGS) technique offers much potential for analyzing thousands of viral sequences from a given host, noticeably improving our ability to quantify within-host sequence diversity in viral infections (Nelson and Hughes, 2015). As an alternative technique, the NGS has been widely applied to estimate the exceptionally-high diversity within viral quasispecies. By means of it, single-nucleotide polymorphisms (SNPs) in various viruses can be systematically analyzed for revealing their evolutionary dynamics (Campo et al., 2014; Hasing et al., 2016; Ni et al., 2016; Yang et al., 2018; Liu et al., 2021).

The purine nucleoside analog, ribavirin, has a broad-spectrum activity against viruses (Graci and Cameron, 2006), including CDV (Elia et al., 2008; Carvalho et al., 2014; Lanave et al., 2017). Ribavirin can exert antiviral activity by increasing the error rate of viral genome replication. Consequently, a gradual accumulation of lethal mutations would lead to a dramatic reduction in replication ability of viral progenies due to error catastrophe. Previous studies demonstrated that RNA viruses cultured in ribavirin-treated cells could evolve to generate ribavirin-resistant variants, characterized by increased fidelity in their RdRps (Pfeiffer and Kirkegaard, 2003; Zeng et al., 2013; Tian and Meng, 2016; Griesemer et al., 2017; Li et al., 2019). Therefore, the ribavirin is an ideal mutagen used to screen for high-fidelity variants for revealing a key molecular mechanism in viral RdRps.

Reverse genetics technique is broadly used to rescue recombinant CDVs for expressing foreign proteins, *e.g.*, enhanced green fluorescence protein (eGFP). Recombinant eGFP-tagged CDV has been demonstrated to be a useful tool for tracing virus infection (Ludlow et al., 2012). We have previously constructed two reverse genetics platforms for different CDV strains. Both platforms facilitate recovery of marker-tagged (Liu et al., 2020) or antigen-expressing (Liu et al., 2021) recombinant CDV. In this study, a recombinant eGFP-tagged CDV (rCDV-eGFP) was rescued, identified, characterized and then used as a model virus for extra forty passages separately in ribavirin- and non-treated cells. The NGS technique was used for uncovering profiles of genomic mutation in ribavirin- and non-screened progenies.

## Materials and Methods

### Cells, Plasmids, and Virus

BSR-T7/5 and Vero-Dog-SLAM (VDS) cell lines, kindly provided by the China Animal Health and Epidemiology Center, were cultured at 37°C with 5% CO_2_ in Dulbecco’s modified Eagle’s medium (DMEM) supplemented with 10% fetal bovine serum, and containing penicillin (100 U/mL), streptomycin (100 µg/mL), amphotericin B (0.25 µg/mL) and G418 (500 µg/mL). Four plasmids, the rCDV-NLuc cDNA clone, pCAGGS-N, pCAGGS-P and pCAGGS-L, were constructed previously in our laboratory (Liu et al., 2020). A wild-type CDV (wt-CDV), QN strain, was propagated in VDS cells.

### Plasmid Construction and Virus Rescue

**Figure 1:**
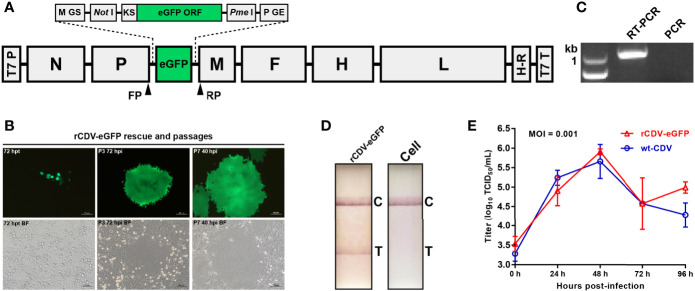
Construction, identification and characterization of rCDV-eGFP. Schematic representation of rCDV-eGFP cDNA clone **(A)**. The proportion of elements does not exactly match them. GS, gene start; GE, gene end; KS, Kozak sequence; ORF, open reading frame; T7 P, T7 promoter; H-R, hepatitis delta virus ribozyme; T7 T, T7 terminator. Forward primer (FP)- and reverse primer (RP)-targeted sites are marked with arrowheads. Rescue and passaging of rCDV-eGFP **(B)**. BF, bright field. Detection of P7 rCDV-eGFP by RT-PCR **(C)**. PCR is designed as a control using the same primers. Detection of rCDV-eGFP- and non-infected cell cultures by test strips **(D)**. T, test; C, control. Multi-step growth curves of the P7 rCDV-eGFP and the wt-CDV in VDS cells during 96-hpi period **(E)**.

The genome sequence of CDV 5804P strain (Genbank access No.: AY386316) (von Messling et al., 2003) had been used to design the rCDV-NLuc cDNA clone for rescuing the NLuc-tagged recombinant CDV (Liu et al., 2020). To construct an rCDV-eGFP cDNA clone, the rCDV-NLuc cDNA clone was modified through replacing its NLuc ORF with that of eGFP (Genbank accession No.: KY295913) using the In-Fusion^®^ Cloning kit (Takara, Dalian, China). The rCDV-eGFP cDNA clone, schematically shown in **Figure 1A**, was purified using the PureLink™ HiPure Plasmid Maxiprep Kit (Thermo Fischer, Carlsbad, USA) for rescuing the rCDV-eGFP. Briefly, BSR-T7/5 cells (Buchholz et al., 1999) were seeded into a six-well plate. A cell monolayer at 70% confluency was co-transfected with the rCDV-eGFP cDNA clone (4 µg/well), pCAGGS-N (2 µg/well), pCAGGS-P (1 µg/well) and pCAGGS-L (1 µg/well) using Lipofectamine 2000 (Thermo Fisher, Carlsbad, USA) according to the manufacturer’s instruction. Co-transfected cells were cultured for 72 h, and then digested with trypsin for further co-cultivation with VDS cells. The rescued rCDV-eGFP was subjected to serial blind passages in VDS cells.

### RT-PCR Analysis of rCDV-eGFP

The rCDV-eGFP was harvested at passage-7 (P7) to extract viral RNA for RT-PCR analysis using the PrimeScript™ High Fidelity One Step RT-PCR Kit (Takara, Dalian, China). The forward primer (FP: 5’-gatcaaaagtatcacacatgcttaa-3’) targeted the 3’-end sequence of P ORF; the reverse primer (RP: 5’-gatcgaagtcgtacacctcagtcat-3’) targeted the 5’-end sequence of M ORF (**Figure 1A**). The RT-PCR reaction underwent 45°C for 10 min, 94°C for 2 min and then 30 cycles at 98°C (10 s), 55°C (15 s) and 68°C (10 s). The P7 RNA sample was also subjected to PCR analysis using the FP/RP to eliminate the possibility of plasmid residues affecting RT-PCR analysis. The PCR contained 2 × PrimeSTAR Max Premix (Takara, Dalian, China), and underwent 30 cycles at 98°C (10 s), 55°C (10 s) and 72°C (10 s). RT-PCR and PCR products were detected by agarose gel electrophoresis, followed by Sanger sequencing of the RT-PCR product.

### Test Strip Detection of rCDV-eGFP

Culture supernatant with rCDV-eGFP-infected cells was harvested at P7 for a single freeze-and-thaw cycle, followed by detection using a test strip of CDV infection (Mensall^®^, Suqian, China), according to the manufacturer’s instruction. Non-infected cell culture was also analyzed as a control.

### Growth Kinetics of rCDV-eGFP

Growth kinetics of the P7 rCDV-eGFP was compared with that of the wt-CDV *in vitro*, as described previously (Liu et al., 2020). Briefly, VDS cells were plated into five 12-well plates (1.5×10^6^ cells/well, and 6 wells/plate) for incubation at 37°C for 2 h. The rCDV-eGFP and wt-CDV was separately inoculated (MOI = 0.001) into all plates (3 wells/sample) for incubation at 37°C for 2 h, and then supernatants were replaced with DMEM for further incubation at 37°C. At 0, 24, 48, 72 and 96 h post infection (hpi), a plate was randomly removed from the incubator, and subjected to a single freeze-and-thaw cycle to collect supernatant for viral titration by TCID_50_ assay. The viral titer for each sample was calculated by the Spearman-Kärber equation (Finney, 1952).

### Extra Forty Serial Passages of rCDV-eGFP

The P7 rCDV-eGFP was independently passaged (3 d/passage) in ribavirin- and non-treated VDS cells for 40 serial passages (P8 to P47) further. The 50% cytotoxic concentration (CC_50_) value of ribavirin had been measured at 6.1 mM for the VDS cell line, and additionally the ribavirin had been determined for a 50% effective concentration (EC_50_) value of 432 µM against another reporter-tagged CDV (5804P strain) (Liu et al., 2020). Therefore, to make the rCDV-eGFP gradually adapt to the ribavirin-treated cells, ribavirin concentration gradually increased in DMEM with passaging: 240 µM from P8 to 13, 320 µM from P14 to 22, 360 µM from P23 to 24, 400 µM from P25 to 29, and 440 µM from P30 to 47. Two rCDV-eGFP progenies, undergoing serial passages in ribavirin- and non-treated VDS cells, were named rCDV-eGFP-R and -N, respectively.

### NGS of rCDV-eGFP-R and -N at P47

Culture supernatants of rCDV-eGFP-R- and -N-infected cells were separately harvested at P47 for extracting total RNAs using the Viral RNA/DNA Extraction Kit (Takara, Dalian, China). The RNA samples were reverse transcribed by random hexamers using the HiScript^®^ 1st Strand cDNA Synthesis Kit (Vazyme, Nanjing, China), according to the manufacturer’s instruction. The Illumina sequencing and library construction were performed as described previously (Liu et al., 2021). In brief, the NEBNext^®^ Ultra^™^ II RNA Library Prep Kit (NEB, Ipswich, MA, USA) was used for library construction. After adapter ligation, ten cycles of PCR amplification were performed for sequencing target enrichment. The libraries were pooled at equal molar ratio, denatured and diluted to optimal concentration prior to sequencing. The Illumina NovaSeq 6000 (Illumina, San Diego, CA, USA) was used for sequencing to generate pair-end 150 bp reads.

### Processing and Analysis of NGS Data

As described previously (Liu et al., 2021), raw reads were filtered by fastp (https://github.com/OpenGene/fastp) to remove sequencing adapters and low-quality reads, including those reads scored < Q20. Ribosomal RNAs and host reads subtraction by read-mapping were performed with BBMap program (https://github.com/BioInfoTools/BBMap)*. De novo* genome assembly was performed using SPAdes v3.14.1 (https://github.com/ablab/spades), as described previously (Nurk et al., 2013). These extracted assembled scaffolds limited the minimum contig length to 100 bases, with the best BLAST hits to the NCBI nucleotide database. High-quality filtered reads were mapped against the full-length sequence of rCDV-eGFP antigenome by Burrows-Wheeler Aligner v0.7.17 (http://bio-bwa.sourceforge.net/), which also generated BAM file to calculate the mapping depth and coverage. SNPs were identified using an integrated software package, Snippy v4.4.5 (https://github.com/tseemann/snippy), which included both substitutions and insertions/deletions. The available SNP results were selected if mapping quality was ≥ 60 and depth was ≥ 10.

## Results

### Competent rCDV-eGFP Is Recovered From Its cDNA Clone

The rCDV-eGFP cDNA clone was co-transfected with three helper plasmids for rescuing the rCDV-eGFP. A small number of plasmid-transfected cells had begun to emit green fluorescence (**Figure 1B**) at 72 h post transfection (hpt). The cell monolayer was digested with trypsin for further co-cultivation with VDS cells. The rescued rCDV-eGFP was subjected to serial blind passages in VDS cells. Fluorescent syncytium formation was always observable during passaging (**Figure 1B**).

### The rCDV-eGFP Is Identified by RT-PCR and Test Strip

Total RNA was extracted from rCDV-eGFP-infected cell culture at P7 for RT-PCR analysis to confirm the viral identity. An expected band of amplicon size (1001 bp) was observed only on the RT-PCR lane (**Figure 1C**). As a control, PCR detection revealed no plasmid residue of cDNA clone affecting RT-PCR analysis (**Figure 1C**). The Sanger sequencing showed that the P7-based RT-PCR product was identical to the 1001-bp-long sequence. Additionally, rCDV-eGFP- and non-infected culture supernatants were detected by test strips, indicating only the former with a positive result (**Figure 1D**).

### The rCDV-eGFP Has Similar Growth Kinetics to That of the wt-CDV

Growth kinetics of rCDV-eGFP at P7 was compared with that of wt-CDV during the 96-h period of viral culture. Syncytia induced by both viruses were observable at 24 hpi, and exacerbated over time. Both viruses exhibited similar growth kinetics during 72 hpi (**Figure 1E**), but a significant difference at 96 hpi.

### NGS Shows Analyzable Sequencing Depths

**Figure 2:**
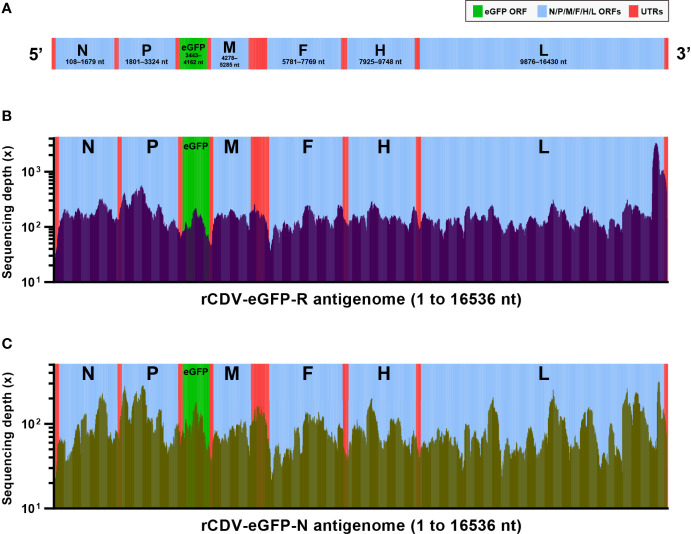
Next-generation sequencing of rCDV-eGFP-R and -N at P47. Schematic representation of rCDV-eGFP antigenome **(A)**. All elements proportionally match their actual lengths in viral antigenome. ORF, open reading frame; UTR, untranslated region. Depth and coverage of next-generation sequencing across the rCDV-eGFP-R **(B)** and -N **(C)** antigenomes. All elements proportionally match their actual lengths in viral antigenome.

The rCDV-eGFP had a 16536-nt-long recombinant genome. **Figure 2A** schematically showed all ORFs and UTRs in proportion to their actual distributions in the viral antigenome. To uncover mutation profiles of rCDV-eGFP-R and -N at P47, viral samples were subjected to NGS analysis. The complete NGS data were deeply analyzed using bioinformatic tools, yielding acceptable sequencing depths. The average depths were 195× and 93× for rCDV-eGFP-R (**Figure 2B**) and -N (**Figure 2C**) antigenomes, respectively. Two samples were determined to have an approximately 99.9% of coverage range across the full-length antigenome sequence. Uncovered regions were located only at 5′- and 3′-end regions in the antigenome.

### NGS Reveals 62 SNMs in rCDV-eGFP-R Antigenome

**Figure 3:**
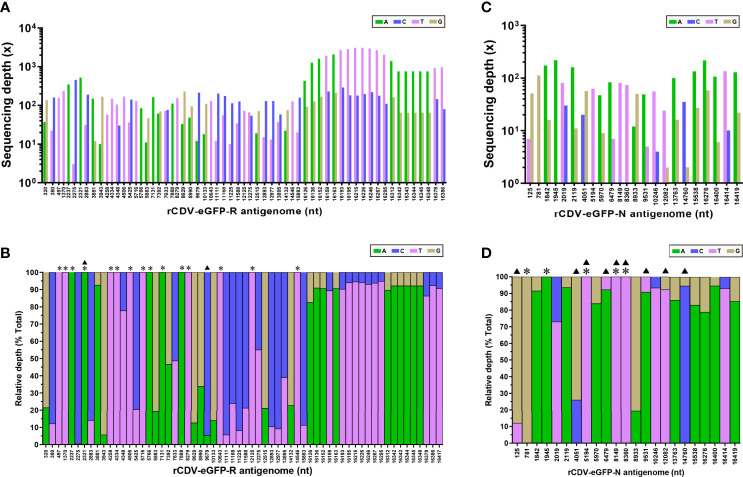
Sequencing depths of NGS for detectable positions with SNM in rCDV-eGFP-R and -N antigenomes at P47. Absolute **(A)** and relative **(B)** sequencing depths of rCDV-eGFP-R. Absolute **(C)** and relative **(D)** sequencing depths of rCDV-eGFP-N. *Relative depth of 100% at a given site. ^▲^Occurrence of transversion at a given site.

**Figure 4:**
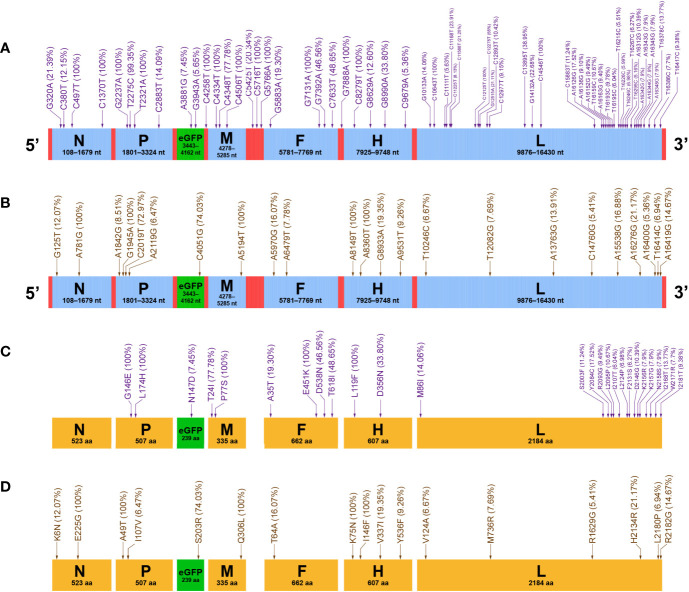
Mutation profiles of rCDV-eGFP-R and -N at P47. Distributions of SNMs in rCDV-eGFP-R **(A)** and -N **(B)** antigenomes. Distributions of SAAMs in rCDV-eGFP-R **(C)** and -N **(D)** proteins. All UTRs and ORFs proportionally match their actual lengths in viral antigenome. All 7 proteins proportionally match their actual lengths. Arrows indicate mutation sites that do not exactly match their definite positions in antigenomes or in proteins. Mutation frequencies are enclosed within brackets.

The NGS analysis showed that a total of 62 SNMs arose in the rCDV-eGFP-R antigenome at P47 (**Supplementary 1**). **Figures 3A, B** showed absolute and relative sequencing depths for all 62 SNMs, respectively. Out of all SNMs, 47 were recognized as SNPs with mixture of two nucleotides, and the others were characterized by total single-nucleotide substitution (TSNS, marked with “*” in **Figure 3B**), namely 100% of mutation frequency. All 62 SNMs were unevenly distributed in the rCDV-eGFP-R antigenome, as schematically shown in **Figure 4A**, in which mutation frequency of each SNM was enclosed within a bracket. There only were two transversions, T2321A and C9679A (**Figure 3B**, triangle-marked), and the other 60 SNMs were transitions, including 36 C/T and 24 A/G ones. Seven ORFs, namely the N (1572 nt), P (1524 nt), eGFP (720 nt), M (1008 nt), F (1989 nt), H (1824 nt) and L (6555 nt), exhibited 4, 4, 2, 3, 4, 4 and 36 SNMs, respectively (**Figure 4A**). Their mutation rates were 0.25%, 0.26%, 0.28%, 0.30%, 0.20%, 0.21% and 0.55%, respectively. The L ORF exhibited a 4-nt-long fragment with complex mutations (A16342G, A16343G, A16344G and A16345G) at a low but non-negligible mutation frequency (7.9%). In addition, three intergenic UTRs, *i.e.*, the eGFP/M, M/F and F/H regions, revealed 1, 3 and 1 SNMs, respectively (**Figure 4A**). Out of these SNMs in UTRs, only one (C5425T) existed as the SNP, and the others showed a mutation frequency of 100%.

### NGS Reveals 23 SNMs in rCDV-eGFP-N Antigenome

A total of 23 SNMs (**Supplementary 2**), including 9 transversions (**Figure 3D**, triangle-marked) and 14 transitions, were identified in the rCDV-eGFP-N antigenome at P47. Absolute and relative sequencing depths for these 23 SNMs were shown in **Figures 3C, D**, respectively. Five SNMs were identified as TSNSs (**Figure 3D**, asterisk-marked). **Figure 4B** schematically revealed the distribution of 23 SNMs with mutation frequency in the rCDV-eGFP-N antigenome. The N, P, eGFP, M, F, H and L ORFs had 2, 4, 1, 1, 2, 4 and 9 SNMs, respectively, whereas SNM was not found in any of the UTRs (**Figure 4B**).

### Twenty-Six Single-Amino Acid Mutations Are Identified in rCDV-eGFP-R

Twenty-eight SNMs led to 26 SAAMs in 6 proteins of the rCDV-eGFP-R at P47 (**Figure 4C**). Out of these 26 SAAMs, 24 directly resulted from their individual SNMs, and the other two (K2156R and K2157G) were attributed to the complex mutations (A16342G, A16343G, A16344G and A16345G) in the L ORF. Mutation frequencies of SAAMs were enclosed within brackets in **Figure 4C**. There were 5 amino acid sites with mutation frequency of 100%. Out of the 7 proteins, only the N protein had no SAAM, and the L protein showed the most SAAMs but all at low mutation frequencies (6.04 to 17.52%). The eGFP as a foreign protein displayed only one SAAM (N147D) in it.

### Seventeen SAAMs Are Identified in rCDV-eGFP-N

The rCDV-eGFP-N ORFs totally harbored 23 SNMs, composed of 17 missense and 6 silent mutations. Therefore, a total of 17 SAAMs are identified in proteins of the rCDV-eGFP-N at P47 (**Figure 4D**). The N, P, eGFP, M, F, H and L proteins exhibited 2, 2, 1, 1, 1, 4 and 6 SAAMs, respectively. There were 5 amino acid sites with mutation frequency of 100%.

## Discussion

A rescue system for CDV was reported as early as 2000 (Gassen et al., 2000). CDV is an effective vector to express foreign proteins (Parks et al., 2002; Plattet et al., 2004; Ludlow et al., 2012; Wang et al., 2012; Liu et al., 2014; Chen et al., 2019). We had established previously the reverse genetics platform of CDV 5804P strain (Liu et al., 2020). In the present study, we used this platform to rescue the rCDV-eGFP, in attempting to use the eGFP as a fluorescent reporter for independently unveiling viral evolutionary patterns under the mutagen- and non-treated circumstances. The rCDV-eGFP had a similar growth curve to that of the wt-CDV during the 72-hpi period (**Figure 1E**), suggesting no significant interference of eGFP with viral growth.

Viral self-proteins are intrinsically expressed in CDV-infected cells. Harmful or even lethal SNMs were an inevitable event with viral passaging, but would not accumulate in viral self-sequences for avoiding the impact of error catastrophe on virus propagation. In contrast, SNMs should be unrestricted in the eGFP ORF during viral replication, owing to the eGFP as a foreign protein, which as such is hardly involved in a series of CDV-related events, *e.g.*, regulation and packaging. SNMs would be random, uncontrolled and retainable in the eGFP ORF during virus growth, therefore initially prompting us to use the rCDV-eGFP to uncover its mutation profile and the viral quasispecies diversity after serial dozens of passages. To promote occurrence of SNMs, rCDV-eGFP-infected cells were treated with the ribavirin, which could act as a viral mutagen forcing RNA viruses into mutagenesis, and even error catastrophe (Cameron and Castro, 2001; Crotty et al., 2001).

The EC_50_ value was 432 µM for the ribavirin against CDV, as determined in our previous report (Liu et al., 2020). In order to make the rCDV-eGFP gradually adapt to selective pressure, the ribavirin concentration progressively increased (240, 320, 360, 400 and 440 µM) with passaging in virus-infected cells. The P7 rCDV-eGFP was subjected to 40 serial passages (P8 to P47) in ribavirin-treated cells. The P47 progeny was speculated to form a rich diversity of viral quasispecies, since most RNA viruses were genetically unstable when they replicate in hosts (Andino and Domingo, 2015). The NGS analysis was used to compare quasispecies diversities between the rCDV-eGFP-R and -N at P47. The reason why the Sanger sequencing was not used here was its inability to quantify the complexity of mutant spectra. Alternatively, the NGS, capable of generating a large dataset to identify SNMs in viral genomes (Huang et al., 2019; Lu et al., 2020), was used in this study.

As a non-self sequence in the recombinant, the eGFP ORF would theoretically contain much more SNMs per 100 nt than viral self-sequences do at P47. Nonetheless, the NGS analysis revealed that the eGFP ORF harbored only two (A3881G and G3943A) and one (C4051G) SNMs in the rCDV-eGFP-R and -N, respectively, suggesting that despite the mutagenic pressure exerted by ribavirin during viral passaging, the eGFP ORF as such did not undergo rich SNMs in the rCDV-eGFP-R antigenome. The G3943A was a silent mutation, whereas the A3881G was a missense mutation, causing an SAAM (N147D) with mutation frequency of 7.45% in the eGFP of rCDV-eGFP-R. It remained to be elucidated whether, to some extent, this SAAM was responsible for one or two fluorescence-attenuated or even -disappeared syncytia that occasionally appeared during passaging (data not shown). Despite one missense mutation (C4051G) found in the eGFP ORF of rCDV-eGFP-N at P47, non-fluorescent syncytia were invisible during viral passages in non-treated cells (data not shown).

The other sequences were simultaneously analyzed for revealing their mutation profiles in the rCDV-eGFP-R and -N at P47. As to the rCDV-eGFP-R, the N, P, M, F and H ORFs were demonstrated to have similar mutation frequencies to that of the eGFP ORF. The L ORF had an approximately 2-fold higher mutation frequency than the other ORFs did in the rCDV-eGFP-R. Interestingly, out of 36 SNMs in the L ORF, 22 congregated in a short region (nt 15883 to 16417) closer to the 3’ end of L gene, implying this region being lowly conserved (**Figure 4A**). In comparison with the rCDV-eGFP-R, the rCDV-eGFP-N showed a low mutation frequency across the full-length antigenome (**Figure 4B**). On the one hand, the rCDV-eGFP-N showed only nine SNMs in its L ORF. On the other hand, a single SNM was not identified in its UTRs. Morbillivirus V protein is produced from its P gene through a frame shift, by the incorporation of one G residue during transcription at a particular mRNA editing site (5’-UUAAAAAGGGCACAG-3’), which is conserved among morbilliviruses (Mahapatra et al., 2003; Muhammad et al., 2013). Indeed, by means of the NGS, we identified this site (data not shown), into which one extra G residue was inserted to form an edited fragment (5’-UUAAAAAGGGGCACAG-3’).

The rCDV-eGFP-R and -N had 62 and 23 SNMs, respectively, confirming our previous speculation that the ribavirin was able to induce error-prone replication of the rCDV-eGFP *in vitro*. Interestingly, the comparative analysis showed no SNM that coexisted in both progenies at P47, suggesting that irrespective of ribavirin-exerted pressure, mutation events randomly occurred during viral passaging. Transitions are the types of mutations associated with ribavirin mutagenesis. Agudo et al. (2010) demonstrated that *via* modulation of transition types, viral adaptation to ribavirin led to extinction-escape (Agudo et al., 2010). Indeed, the present study also exhibited that the rCDV-eGFP-R had a much higher ratio of transition/transversion (60/2, **Figure 3B**) than the rCDV-eGFP-N did (14/9, **Figure 3D**).

The RdRp-caused error rate is normally a primary driving force of mutation frequencies observed in RNA virus populations (Borderia et al., 2016). Morbilliviruses are characterized by high mutation frequencies in their genomes during serial passages *in vitro*, mainly attributed to the lack of effective proofreading activities in their L proteins [see our review in (Liu et al., 2016)]. Measles virus has shown a spontaneous mutation rate of 1.8 × 10^−6^/nt/replication under nonselective conditions (Zhang et al., 2013). An earlier report estimated the measles virus with a mutation rate of 9 × 10^−5^/nt/replication, and with a genomic mutation rate of 1.43/replication (Schrag et al., 1999).

Our previous report revealed that the N ORF of wild-type small ruminant morbillivirus (SRMV) underwent the most mutation events among the six structural genes during 90 serial passages in non-treated VDS cells (Wu et al., 2016). Unfortunately, the data in this report was based on the Sanger sequencing. We subsequently rescued an eGFP-tagged recombinant SRMV (rSRMV-eGFP) (Liu et al., 2019), followed by 45 serial passages in ribavirin-treated VDS cells. More recently, its mutation profiles with serial passaging were uncovered *via* the NGS analysis (Liu et al., 2021), revealing that a total of 34 SNMs, including 5 silent, 21 missense and 1 nonsense mutations, arose with passaging. The L ORF was found to harbor only 8 SNMs, all of which were missense mutations. The eGFP had one nonsense mutation, causing that non-fluorescent syncytia became gradually visible with passaging. Compared with the rSRMV-eGFP that underwent 45 passages with ribavirin screening, the rCDV-eGFP-R in the present study showed a high mutation frequency in its L ORF, but no nonsense mutation in its eGFP ORF.

Historically, a virulence-attenuated CDV (Rockborn strain) was demonstrated to revert back to a virulent status after serial passages in dogs (Appel, 1978). Dogs, vaccinated with a polyvalent vaccine containing the Rockborn strain, exhibited suspected encephalitis. The Rockborn strain was withdrawn from several markets after the mid 1990s (Martella et al., 2011). Considering a potential risk factor for reversion to virulence, it is necessary to screen for high-fidelity CDV strains for exploring their own mechanisms in high-fidelity replication. In the present study, the mutagen-resistant progeny was demonstrated to be with rich quasispecies diversity at P47. According to the classical theory of viral quasispecies (Lauring and Andino, 2010; Domingo et al., 2012; Andino and Domingo, 2015; Borderia et al., 2016), we speculated that the P47 progeny of rCDV-eGFP-R might be composed of high-, moderate- and low-fidelity mutants. If the high-fidelity ones can be purified from such a mutant swarm, key SAAMs in the L protein would clarify a molecular mechanism of viral high-fidelity replication.

## Data Availability Statement

The datasets presented in this study can be found in online repositories. The names of the repository/repositories and accession number(s) can be found below: NCBI (accession: PRJNA752812).

## Author Contributions

FL conducted experiments and wrote the manuscript. NW, JL, QW and YH performed the experimental works. YZ and HS provided the fundings. HS supervised the project. All authors contributed to the article and approved the submitted version.

## Funding

This work was supported by the Shandong Key Research and Development Program (2019GNC106074), and the Open Project Fund of State Key Laboratory of Microbial Technology, Shandong University (M2021-19).

## Conflict of Interest

The authors declare that the research was conducted in the absence of any commercial or financial relationships that could be construed as a potential conflict of interest.

## Publisher’s Note

All claims expressed in this article are solely those of the authors and do not necessarily represent those of their affiliated organizations, or those of the publisher, the editors and the reviewers. Any product that may be evaluated in this article, or claim that may be made by its manufacturer, is not guaranteed or endorsed by the publisher.
